# A Case of Statin-Induced Necrotizing Autoimmune Myopathy

**DOI:** 10.7759/cureus.70852

**Published:** 2024-10-04

**Authors:** Inshya Desai, Shivani K Modi, Abdul Subhan

**Affiliations:** 1 Internal Medicine, Jefferson Einstein Montgomery Hospital, East Norriton, USA

**Keywords:** anti-hmg coa reductase, anti-hmgcr antibody, drug-induced myopathy, immune-mediated necrotizing myopathy, statin-induced

## Abstract

Statins, commonly used for hyperlipidemia and more importantly having proven efficacy in lowering cardiovascular risk, are a very popular class of medications. Side effects are usually mild, and the class as a whole is generally well-tolerated by patients. However, one rare and more serious side effect is statin-induced autoimmune necrotizing myopathy. A 65-year-old woman with a past medical history of hyperlipidemia on atorvastatin for four years, type 2 diabetes, sciatica, and a prior history of endometrial cancer, presented to the emergency department due to proximal muscle weakness worsening over 2-3 months. On labs, her aspartate transaminase (AST) and alanine transaminase (ALT) were both elevated at 344 and 244, respectively. Additionally, creatine kinase (CK) was 13,000, C-reactive protein (CRP) was 42, and aldolase was 80.4. She was antinuclear antibody (ANA) negative but found to be anti-3-hydroxy-3- methylglutaryl-coenzyme A reductase (HMGCR ab) positive. Her magnetic resonance imaging (MRI) showed significant edema in bilateral thighs. During her hospital course, she was started on prednisone, fluids, and intravenous immunoglobulin (IVIG). Once completing two days of IVIG, the patient was discharged to rehab with rheumatology follow-up. The purpose of this case is to elucidate a rare side effect of a medication despite being on it for several years and to increase awareness and attention to an adverse effect that may one day lead to stratifying patients' risk on the medication.

## Introduction

Statins work by inhibiting HMG-CoA reductase, which prevents the conversion to mevalonate. This is the rate-limiting enzyme in the cholesterol biosynthesis pathway [1]. One of the most common side effects experienced by patients is muscle pain and cramps. Muscles in general rely on functioning ryanodine receptors to regulate calcium for muscle contraction and stimulation. The mechanism by which statins can impact muscles focuses on statins interfering with and compromising host ryanodine receptors. This leads to irregular calcium leaks which can subsequently trigger cell death in the muscles leading to cramps [2]. Additionally, a rare side effect is statin-induced autoimmune necrotizing myopathy, which is seen in about one out of every 100,000 patients taking a statin. There is no truly identified correlation with the onset of symptoms and severity of disease with how long the patient has been on the medication [3]. The antibody disrupted in this disease process is 3-hydroxy-3-methyl-glutaryl-coenzyme A reductase (HMGCR) [4]. While there are several postulated mechanisms about how statins can affect the muscle and cause disruption, they are not thought to act alone but rather in conjunction with each other, making the pathogenesis not yet fully understood. Here, we present a case of statin-induced myonecrosis in patient who had been on a statin for years but quickly developed progressive proximal weakness and myonecrosis.

## Case presentation

**Table 1:** Patient's lab values compared with normal values AST: Aspartate transaminase; ALT: alanine transaminase

Lab name	Patient's lab values	Normal values
AST	344 u/l	8-33 u/l
ALT	244 u/l	4-36 u/l
Creatinine	1.37 mg/dL	0.6-1.1 mg/dL
Blood urea nitrogen (BUN)	28 mg/dL	6-24 mg/dL
Creatinine kinase (CK)	13204 u/l	30-135 u/l
C-reactive protein (CRP)	42.6 mg/dL	<0.3 mg/dL
Erythrocyte sedimentation rate (ESR)	102 mm/hr	0-33 mm/hr
Antinuclear antibody (ANA)	Negative	Negative
Aldolase	80.4 u/l	1-7.5 u/l
HMGCR antibody	184 CU/ml	<14.9 CU/ml

**Figure 1:**
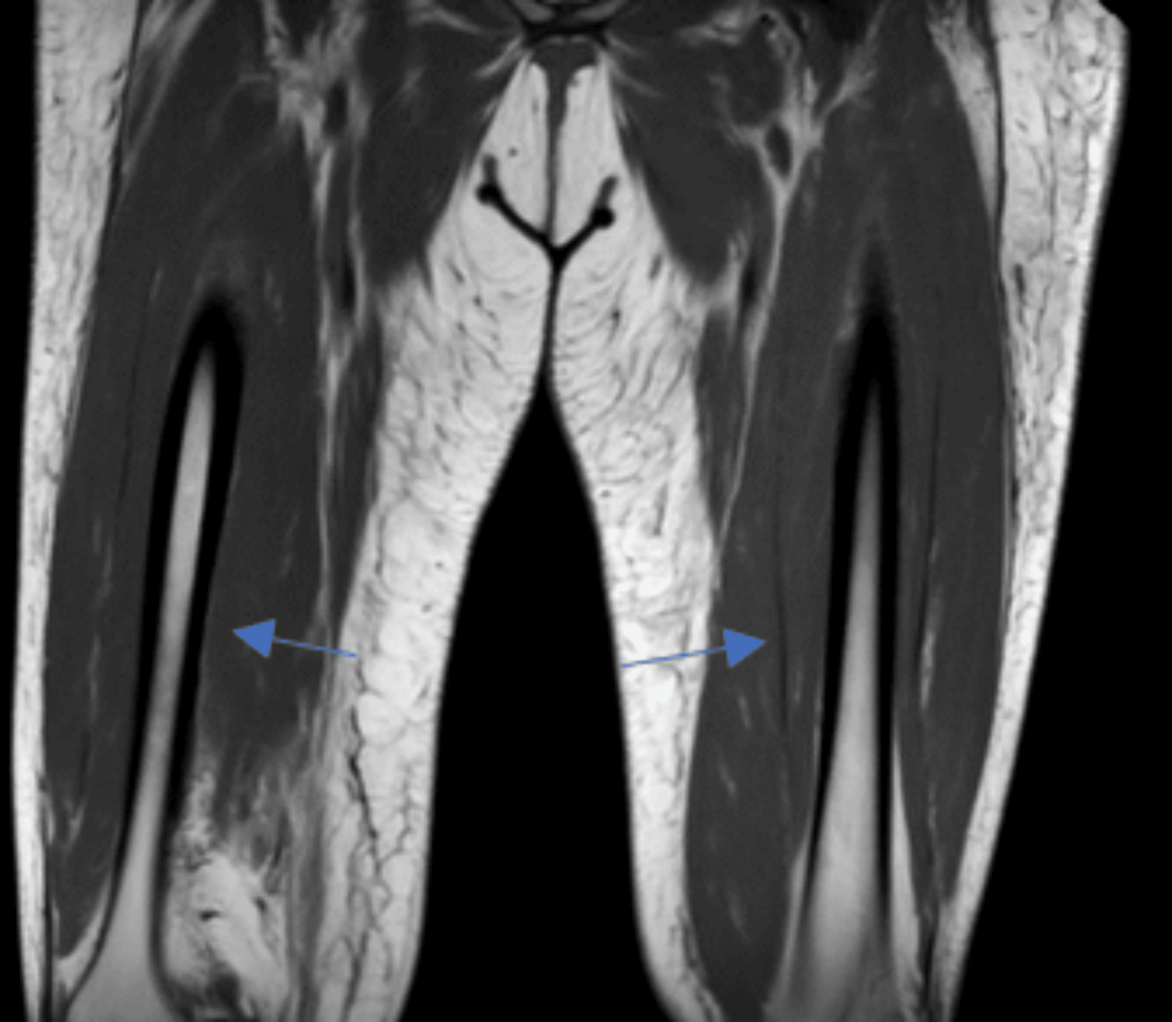
T1-weighted MRI of bilateral thighs showing the presence of edema

**Figure 2:**
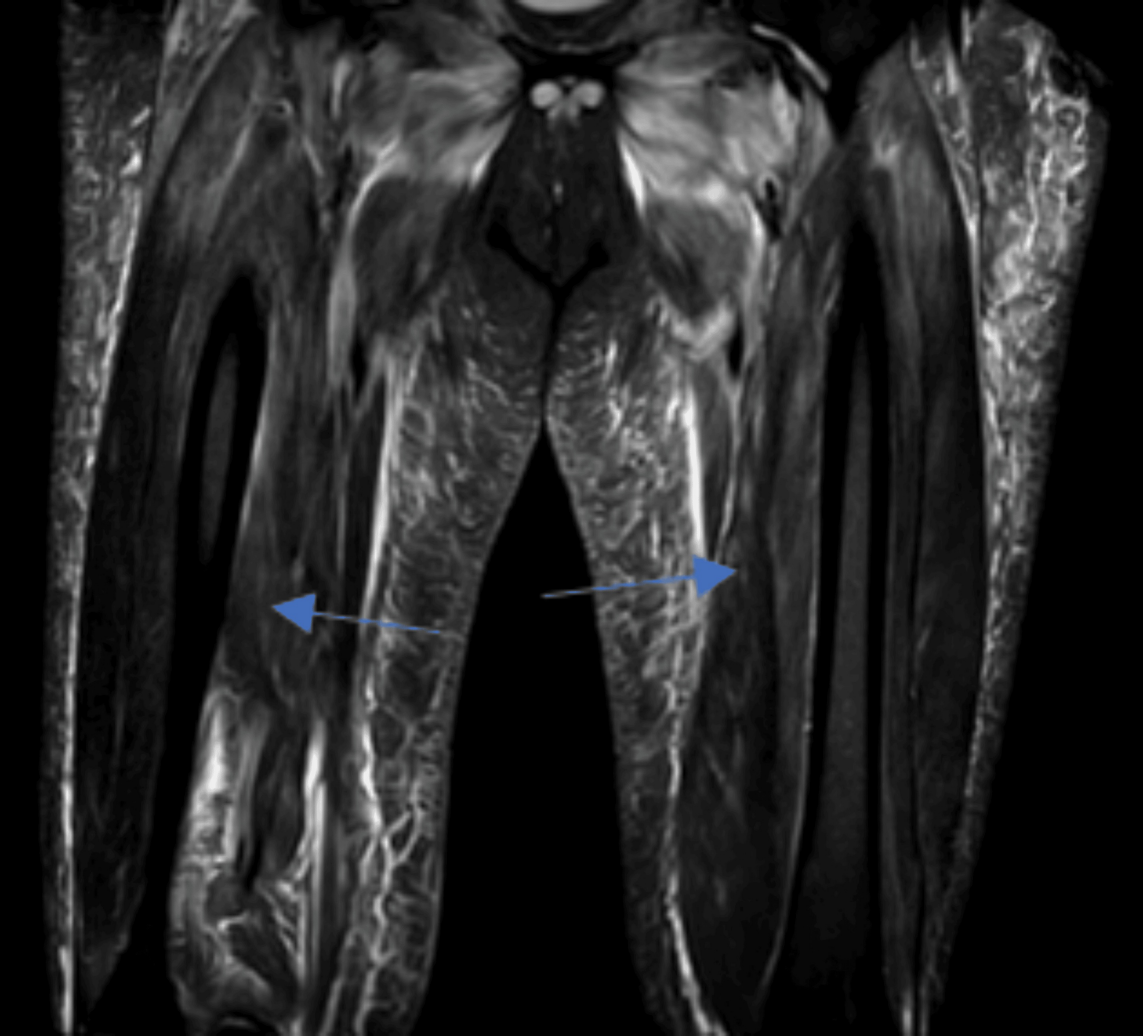
T2-weighted MRI showing the presence of edema in bilateral thighs

**Figure 3:**
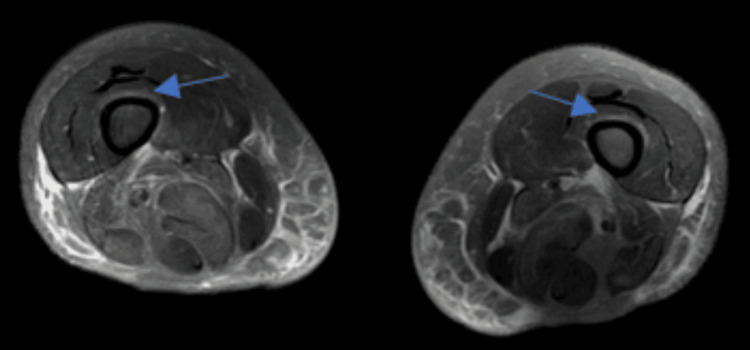
Axial view of the bilateral thighs showing edema around the proximal muscles

A 65-year-old female with a past medical history of hyperlipidemia, type 2 diabetes, sciatica, and a prior history of endometrial cancer presented to the emergency department due to proximal muscle weakness worsening over two months. She was having trouble performing her activities of daily living (ADLs); she needed assistance with getting in and out of bed and getting in and out of the shower. She also felt that over the past couple of months, when she was walking, she felt she had sandbags on her legs. She denied any fever, chills, rashes, joint pain, or initiating events. She denied any family history of autoimmune disorders. Her only outpatient medications included metformin 1000 mg twice daily and rosuvastatin 20 mg which she had been on for about four years. Her physical exam was significant for left-sided weakness 2/5 in the deltoid and elbow, with preserved strength in the wrist. On the right side, she was 3/5 in the deltoid, 4/5 in the right elbow, and 5/5 in the wrist. Lower extremity was 5/5 with deep tendon reflexes diminished throughout. Cardiac exam and pulmonary exam were normal, along with normal skin findings. On laboratory studies, aspartate transaminase (AST) was 344 u/l and alanine transaminase (ALT) was 244 u/l, creatinine (Cr) was 1.37 mg/dL, blood urea nitrogen (BUN) was 28 mg/dL, creatinine kinase (CK) was 13204 u/l, C-reactive protein (CRP) was 42.6 mg/dL, and erythrocyte sedimentation rate (ESR) was 102 mm/hr. Her antinuclear antibody (ANA) was negative, and aldolase was 80.4 u/l. Her HMGCR antibody was 184 CU/ml, and other myositis panel was negative (Table 1). The patient was initially admitted to the general floors with a diagnosis of statin-induced rhabdomyolysis based on the elevated CK. The patient was started on aggressive intravenous hydration, and her rosuvastatin was discontinued. With fluids alone, CK down trended to 8000, but subsequently increased to 11000 and then continuously and steadily decreased to 7000, 6000, 5000, 4000, and then 3000. On day three of her stay, the patient was also started on prednisone to help with some of the inflammation. Fluids were stopped on day five due to fluid overload and subsequent development of lower extremity edema. MRI of bilateral lower extremity was also done during the course of her hospitalization (Figures 1-3). On day nine of the patient's hospital course, HMGCR antibody came back positive and aldolase was high as well. The patient was started on 75 g intravenous immunoglobulin (IVIG) for two days and then followed up with rheumatology outpatient. She was seen in the office and is following a monthly IVIG infusion for six months with reassessment. The patient additionally was in physical therapy for weeks following her admission to help rebuild the strength she had been losing for months.

## Discussion

Statins have shown great efficacy in reducing cardiovascular events in patients with high cholesterol; they are one of the most widely used medications, with millions of people taking it daily. Side effects of myalgias are well-documented throughout; however, a rare side effect of myonecrosis only affects about 0.01% of those taking a statin [5].

The diagnosis of statin-induced necrotizing autoimmune myopathy (SINAM) is characterized by proximal muscle weakness and elevated serum Cr. Imaging may also show the presence of edema in the muscles. Another characteristic marker of SINAM is the presence of anti-HMGCR CoA antibody which is not seen in any other necrotizing myopathy, making this highly sensitive and specific to the disease. Additionally, when the diagnosis is unclear, biopsies may aid by showing histological evidence of necrosis, with little or no evidence of inflammatory cell infiltration. However, biopsy is not mandatory for diagnosis due to high sensitivity and specificity of the HMGCR antibody. Anti-HMGCR is not the only antibody associated with SINAM, the presence of anti-signal recognition particle (SRP) antibody may also lead to the diagnosis, but this is less commonly seen and tested for. SRP can also be associated with cardiomyopathies and more severe muscular atrophies making it less sensitive and specific for the disease [6,7].

The pathophysiology of statin-induced myonecrosis is not fully known; however, there are various proposed mechanisms. One of the most widely accepted mechanisms is that the HMG-CoA reductase acts as its own autoantigen. The idea here is that there is buildup of HMG-CoA reductase due to statin inhibition, which in turn directly causes injury and toxicity to the muscles, leading to the uncomfortable symptoms of muscle cramps. Another proposed mechanism is that the accumulation of HMG-CoA reductase causes conformational change within the HMGC receptor leading to the generation of cryptic epitopes. This accumulation is thought to result in aberrant processing by the antigens which leads to subsequent production of cryptic epitopes, both of which, together, can provoke an intense autoimmune response. It is widely accepted that human leukocyte antigens (HLA) play a large role in autoimmunity and that might be the case in this disease process as well. There is some thought in SINAM; certain peptides produced and derived from HMGCR protein can be more immunogenic eliciting a more robust response leading to autoimmunity [8]. The belief is that these mechanisms of statin-induced myonecrosis are not thought to work independently, but rather concomitantly, which makes the pathogenesis difficult to fully understand. The mainstay of treatment for this disease process is glucocorticoids and cessation of statin medications indefinitely. Additionally, with long-term use of steroids, all patients should be supplemented with calcium, vitamin D, and *Pneumocystis jirovecii *(PJP) prophylaxis [9]. Other treatments can include immunosuppressants and maintenance therapy with mycophenolate mofetil, methotrexate, and azathioprine. Response to treatment is generally monitored through CK levels. Early diagnosis and recognition seem to be key in mitigating potential irreversible consequences of statin-induced autoimmune myonecrosis; with early detection, patients can regain their full functional capacity and lead a normal, healthy life [10].

## Conclusions

This research is important because of the lack of data published due to the rare nature of this disease. Hopefully, by having more research, there might be a chance to stratify those at risk of the disease and severity of the disease. Additionally, it is important to emphasize that the lack of a temporal relationship between statin initiation and symptoms can delay diagnosis leading to more severe symptoms and inability to return to baseline muscle function.
